# Management of Burn Injuries in Brazil by Total Body Surface Area Affected

**DOI:** 10.1002/wjs.70275

**Published:** 2026-02-22

**Authors:** Paulo Henrique Moreira Melo, João Oliveira Góes Neno, Cynthia Florencio de Mesquita, Sarah Lopes Salomão, Lauren Kratky, David P. Mooney, Cristina Pires Camargo

**Affiliations:** ^1^ Faculty of Medicine and Health Sciences Department of Surgery McGill University Montreal Quebec Canada; ^2^ Estácio de Sá University/IDOMED Vista Carioca Campus Rio de Janeiro Rio de Janeiro Brazil; ^3^ Federal University of Pernambuco (UFPE) Recife Pernambuco Brazil; ^4^ Postgraduate Program in Rehabilitation Sciences Hospital for Rehabilitation of Craniofacial Anomalies University of São Paulo (HRAC‐USP) Bauru São Paul Brazil; ^5^ Program in Global Surgery and Social Change Harvard Medical School Boston Massachusetts USA; ^6^ Department of Surgery Dartmouth‐Hitchcock Medical Center Lebanon New Hampshire USA; ^7^ Boston Children's Hospital Harvard Medical School Boston Massachusetts USA; ^8^ Laboratory of Microsurgery and Plastic Surgery School of Medicine, Universidade de São Paulo São Paulo São Paulo Brazil

**Keywords:** burn care delivery, burns, LMIC, plastic surgery, surgery rate

## Abstract

**Introduction:**

Major Burns are life‐threatening injuries that cause approximately 2500 deaths per year in Brazil. The Brazilian healthcare system has 80 hospitals with Burn Care Units (BCUs). However, non specialized hospitals also manage major burn injuries. The aim of this study was to evaluate differences in burn management and outcomes between BCUs and hospitals without BCUs.

**Methods:**

Patients with an ICD‐10 code for a burn injury were identified in a Brazilian discharge database (DATASUS) from 2015 to 2023, were categorized by total body surface area (TBSA). Hospitalizations were compared based on whether they occurred in a BCU or not, adjusting for TBSA, age, and sex.

**Results:**

From 2015 to 2023, there were 22,627 burn injury hospitalizations in Brazil. Of these, 14,187 (62.71%) were treated in Burn Care Units (BCUs), 6553 (28.96%) in non‐BCU hospitals with plastic surgery services, and 1887 (8.34%) in non‐BCU hospitals without plastic surgery. Mean TBSA was highest in non‐BCUs with plastic surgery (42.0%) compared to BCUs (35.2%) and non‐BCUs without plastic surgery (34.4%; *p* < 0.001). Mortality occurred in 9.87% of hospitalizations at BCUs, 7.78% at non‐BCUs with plastic surgery, and 4.45% at non‐BCUs without plastic surgery. After adjusting for TBSA, age, and sex, non‐BCU facilities showed lower odds of mortality (non‐BCU with plastic surgery: aOR 0.49, 95% CI 0.44–0.56; non‐BCU without plastic surgery: aOR 0.36, 95% CI 0.28–0.47; both *p* < 0.001). Transfers to other facilities occurred in 1.28% (182/14,187) of BCUs, 16.26% of non‐BCUs with plastic surgery, and 29.61% (559/1887) of non‐BCUs without plastic surgery. Surgical management rates were highest in BCUs, intermediate in non‐BCUs with plastic surgery, and lowest in non‐BCUs without plastic surgery.

**Conclusion:**

Hospitalizations in BCUs were associated with lower transfer rates and higher rates of surgical intervention. However, BCUs also reported higher mortality rates, suggesting that they likely provided care to the most severely injured burn patients until their final outcomes, whether recovery or death. Despite non‐BCUs with plastic surgery treating burns with higher mean TBSA, BCUs had higher mortality, suggesting that BCUs receive patients with unmeasured risk factors beyond TBSA and serve as referral centers for the most complex cases.

## Graphical abstract

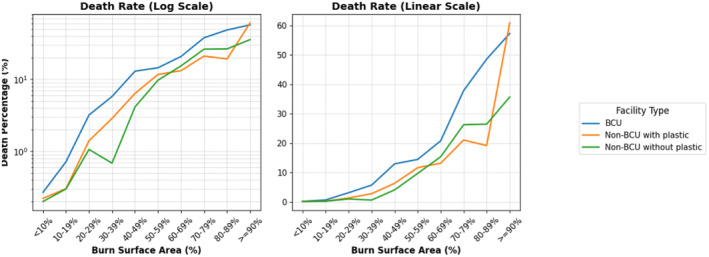

## 1 Introduction

Burn injuries are a significant public health issue worldwide, associated with substantial morbidity and mortality [1]. Approximately 90% of burn‐related deaths occur in low‐ and middle‐income countries (LMICs) [2]. Surgical access is critical for moderate‐to‐major burn patients, as timely and adequate intervention can improve outcomes, especially for those requiring intensive, multimodal management [3]. However, significant gaps remain in the availability of burn care units, trained personnel, and essential equipment, with the World Health Organization estimating 180,000 deaths from burns every year, with the vast majority of them occurring in LMICs [4].

The plastic surgery workforce plays a crucial role in addressing the unmet burden of burn care. However, plastic surgeon workforce density varies significantly across income levels, with LMICs experiencing severe shortages. A study across 15 low‐income countries (LICs) identified only 63 plastic surgeons total, with some countries having no plastic surgeons [5]. According to the 2023 Medical Demographics, Brazil has a plastic surgeon workforce density of 3.67 specialists per 100,000 population [6].

Despite this relatively high plastic surgeon density [7], burn care in Brazil faces significant challenges, with nearly 80% of burn deaths occurring out of hospital, highlighting the need for coordinated efforts to improve care [8].

Brazil has integrated burn care into the national healthcare strategy through a network of facilities known as Burn Care Units (BCU). These play a crucial role in the management and referral of patients with major burns, providing comprehensive care through multidisciplinary teams consisting of surgeons, anesthesiologists, physical therapists, and psychologists [9]. They allow for coordinated efforts to stabilize severely injured patients, provide surgical intervention, and offer long‐term care including rehabilitation. According to the Brazilian Society of Burns (BSB), there are 80 registered BCUs throughout Brazil [10]. However, disparities persist, as BCUs are unevenly distributed, with most located in urban areas in the South and Southeast regions of Brazil, leaving rural populations underserved [11]. Patients living in rural areas without BCUs often receive care in general hospitals lacking plastic surgeons and other specialized resources that improve outcomes in burn care.

In the context of uneven resource distribution, a well‐integrated healthcare system should prioritize treating the most severe cases in hospitals with the greatest availability of specialized resources. In this study, we investigate the management of burn injuries by analyzing hospitalization severity and comparing differences in care delivery and outcomes between Burn Care Units (BCUs) and non‐specialized facilities, as well as between hospitals with and without plastic surgery services.

## 2 Methods

### 2.1 Study Design and Population

This cross‐sectional study analyzes hospitalizations for burn injuries treated in Brazil's publicly funded hospital system Sistema Unico de Saude (SUS) between 2015 and 2023. The study included only the cases where burn severity was classified by total body surface area (TBSA) using ICD‐10 codes T31.0 (burns covering ≤ 10% of the body) through T31.9 (burns covering ≥ 90% of the body).

### 2.2 Data Sources

This study analyzed data from two Brazilian public healthcare databases. Hospitalization records were obtained from the Brazilian Hospital Information System (SIH‐SUS), a national database managed by the Department of Informatics of the Unified Health System (DATASUS). SIH‐SUS provides anonymized demographic, clinical, and procedural details for all publicly funded hospitalizations. To assess human resource availability at the hospital, we incorporated the National Registry of Health Establishments (CNES), a Ministry of Health database that lists all job contracts of all medical specialists in Brazil. Data extraction, integration, and analysis were conducted using R Studio (version 2022.07) with the microdatasus package, a tool designed for Brazilian public health datasets [12].

### 2.3 Eligibility Criteria

#### 2.3.1 Study Parameters and Outcomes

Hospitals were classified based on their capacity to treat burn patients, and facilities designated as Burn Care Units were categorized accordingly. Additionally, hospitals were classified based on whether they had a plastic surgeon on‐site. Hospitalizations were analyzed based on immediate outcomes, estimated hospitalization costs, the types of procedures performed, and the etiology of the burns.

Outcomes were classified based on the SIH‐SUS variable “COBRANÇA,” which indicates the reason for the hospitalization's conclusion. We considered the following outcomes: “Death,” “Leaving Against Medical Advice (LAMA),” and “Transfer to another facility.” All other outcomes (e.g., improvement, cure, discharge with scheduled outpatient follow‐up) were grouped as “Safe discharge” in this study.

#### 2.3.2 Data Analysis

Descriptive statistics were utilized to summarize the data, presenting counts and percentages for categorical variables, and the median and interquartile range for numeric variables. The Chi‐square test was applied to assess the differences between categories for categorical outcomes. Multivariable logistic regression was performed to evaluate the association between facility type and in‐hospital mortality, adjusting for burn severity (TBSA as a continuous variable, per 10% increase), patient age (per 10‐year increase), and sex. Adjusted odds ratios (aOR) with 95% confidence intervals (CI) were calculated. Stratified analyses were conducted within burn severity categories (< 20%, 20%–39%, 40%–59%, ≥ 60% TBSA) to assess the consistency of associations across different injury severities. Statistical analyses were carried out using Python (statsmodels package) and the IBM SPSS Statistics program version 22.0 Armonk, NY: IBM Corp.

#### 2.3.3 Ethical Aspects

The study was waived IRB approval by Boston Children's Hospital (Protocol IRB‐P00049892) as it uses an anonymous public database without private identifiable information or interaction with individuals. In accordance with Brazilian regulations [13], research using publicly available anonymized data does not require local ethics committee approval.

## 3 Results

### 3.1 Overall

We analyzed public records from 2015 to 2023 on 22,627 burn injuries in Brazil that were classified by total body surface area (TBSA). The mean patient age was 30.8 years (SD 20.7), with 63.4% (14,337/22,627) male patients.

The geographic distribution of BCUs showed strong regional concentration: the Southeast accounted for 45 units (56.25% of the total), followed by the Northeast with 16 (20.00%), the South and Central‐West with 7 each (8.75% respectively), and the North with 5 (6.25%). Plastic surgeons were available on‐site in 100% of BCUs while only 77.64% of patients hospitalized at non‐BCUs were seen by a plastic surgeon during their stay.

Among 22,627 burn cases analyzed in Brazil, 14,187 (62.71%) were managed in the country's 80 specialized Burn Care Units (BCUs), 6553 (28.96%) received care in non‐BCU hospitals with plastic surgery services available, and 1887 (8.34%) were treated in non‐BCU hospitals without access to plastic surgery.

The distribution of burn severity varied significantly across facility types (*χ* ^2^ = 584.4, *p* < 0.001). Mean TBSA was highest in non‐BCU hospitals with plastic surgery (42.0% ± 21.5%), followed by BCUs (35.2% ± 22.1%) and non‐BCU hospitals without plastic surgery (34.4% ± 20.6%; ANOVA *F* = 234.2, *p* < 0.001). Non‐BCU hospitals with plastic surgery treated the highest proportion of severe and critical burns (≥ 40% TBSA): 51.8% compared to 40.1% in BCUs and 34.0% in non‐BCUs without plastic surgery. Minor burns (< 20% TBSA) were most common in non‐BCUs without plastic surgery (25.5%) and BCUs (24.7%), compared to only 12.0% in non‐BCUs with plastic surgery.

### 3.2 Outcomes

**FIGURE 1:**
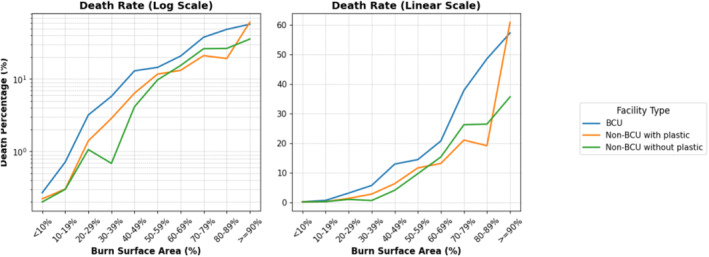
This figure illustrates death rates by burn surface area (%) across facility types. Burn Care Units (BCUs) consistently treated patients with more extensive burns, reflected in higher mortality at greater TBSA levels.

**FIGURE 2:**
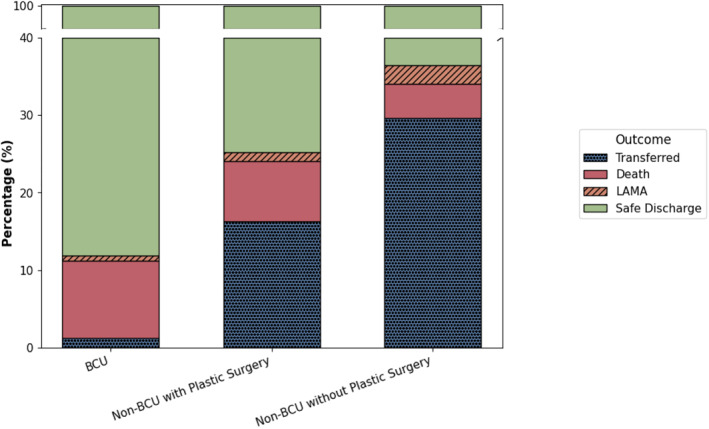
This figure illustrates the distribution of discharge outcomes across facility types. BCUs showed the lowest rates of transfers and LAMA, suggesting greater capacity to manage severe burns without referral.

Mortality rates were higher in BCUs (9.87% [1401/14,187]) compared to non‐BCU hospitals with plastic surgery (7.78% [510/6553]) and non‐BCU hospitals without plastic surgery (4.45% [84/1887]; *p* < 0.0001), even after adjustment for burn extent (Figure 1). Transfers to other facilities occurred in 1.28% (182/14,187) of cases in BCUs, 16.26% (1065/6553) in non‐BCU hospitals with plastic surgery, and 29.61% (559/1887) in non‐BCU hospitals without plastic surgery (*p* < 0.0001, respectively). Safe discharge was achieved in 88.13% (12,503/14,187) of patients hospitalized at BCUs, 74.85% (4904/6553) at non‐BCUs with plastic surgery, and 63.62% (1200/1887) at non‐BCUs without plastic surgery (*p* < 0.0001). Leaving against medical advice (LAMA) occurred in 0.71% (101/14,187) of hospitalizations at BCUs, 1.13% (74/6553) at non‐BCUs with plastic surgery, and 2.33% (44/1887) at non‐BCUs without plastic surgery (*p* < 0.0001) (Figure 2).

**FIGURE 3:**
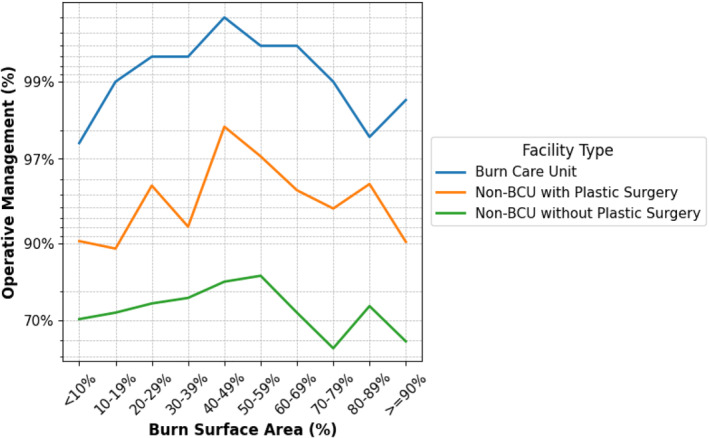
This figure illustrates operative management rates by burn surface area (%) across facility types. Surgical intervention was most frequent in BCUs, with a clear gradient favoring specialized care.

Surgical care was performed in 99.02% (14,048/14,187) of patients hospitalized at BCUs, compared to 94.93% (6221/6553) at non‐BCUs with plastic surgery and 76.40% (1442/1887) at non‐BCUs without plastic surgery (*p* < 0.0001). Across all categories of burn extent, operative management rates remained highest in BCUs, followed by non‐BCUs with plastic surgery and then non‐BCUs without plastic surgery (Figure 3).

### 3.3 Multivariable Analysis

In multivariable logistic regression adjusting for TBSA, age, and sex, hospitalization in non‐BCU facilities was associated with significantly lower odds of mortality compared to BCUs (Table 1). Non‐BCU hospitals with plastic surgery services had adjusted odds ratio (aOR) of 0.49 (95% CI: 0.44–0.56, *p* < 0.001), and non‐BCU hospitals without plastic surgery had aOR of 0.36 (95% CI: 0.28–0.47, *p* < 0.001). Each 10% increase in TBSA was associated with 80% higher odds of mortality (aOR 1.80, 95% CI: 1.75–1.84, *p* < 0.001), and each 10‐year increase in age was associated with 49% higher odds (aOR 1.49, 95% CI: 1.45–1.53, *p* < 0.001). Male sex was associated with lower odds of mortality (aOR 0.77, 95% CI: 0.69–0.86, *p* < 0.001).

**TABLE 1:** Multivariable logistic regression for in‐hospital mortality.

Variable	Crude OR (95% CI)	Adjusted OR (95% CI)	*p*‐value
Facility type			
BCU (reference)	1.00	1.00	—
Non‐BCU with plastic surgery	0.77 (0.69–0.86)	0.49 (0.44–0.56)	< 0.001
Non‐BCU without plastic surgery	0.43 (0.34–0.53)	0.36 (0.28–0.47)	< 0.001
Covariates			
TBSA (per 10% increase)	—	1.80 (1.75–1.84)	< 0.001
Age (per 10 years)	—	1.49 (1.45–1.53)	< 0.001
Male sex (ref: Female)	—	0.77 (0.69–0.86)	< 0.001

*Note: N* = 22,627; Deaths = 1995 (8.8%); Model Pseudo *R* ^2^ = 0.273.

Abbreviations: BCU, Burn Care Unit; CI, Confidence Interval; OR, Odds Ratio; TBSA, Total Body Surface Area.

Stratified analysis by burn severity showed consistently higher mortality in BCUs across all severity categories. Among patients with moderate burns (20%–39% TBSA), mortality was 4.2% in BCUs versus 1.9% in non‐BCUs with plastic surgery and 0.9% in non‐BCUs without plastic surgery. Among patients with critical burns (≥ 60% TBSA), mortality was 36.9% in BCUs versus 23.5% in non‐BCUs with plastic surgery and 25.4% in non‐BCUs without plastic surgery.

## 4 Discussion

This study evaluated hospitalization patterns, management strategies, and patient outcomes across three categories of healthcare institutions treating burn injuries in Brazil: BCUs, non‐BCUs with plastic surgery services, and non‐BCUs without plastic surgery. Our findings demonstrate that while non‐BCU hospitals with plastic surgery treated the most severe burns by TBSA (mean 42.0%), BCUs had higher mortality rates (9.87% vs. 7.78%). Importantly, multivariable analysis adjusting for TBSA, age, and sex showed that this mortality difference persisted and even became more pronounced after adjustment, suggesting that unmeasured factors beyond burn extent contribute to the observed differences.

Burn Care Units (BCUs) and non‐BCU hospitals with plastic surgery services reported higher mortality rates compared to facilities without plastic surgery services, while simultaneously exhibiting lower transfer rates. This pattern likely reflects a greater capacity to manage complex cases without referral. In contrast, non‐BCU hospitals without plastic surgeons relied more heavily on conservative management. These findings highlight the critical role of specialized surgical expertise in guiding treatment decisions, as excessive reliance on conservative approaches may delay wound healing, increase the risk of infection, and contribute to hypertrophic scarring [14, 15].

In LMICs higher burn mortality rates are often associated with delayed specialized care, inadequate fluid treatment, and a lack of surgical expertise [2, 3]. In Brazil, while the public healthcare system provides broad access to treatment, burn care services are unevenly distributed across the country. This study found that hospitals without plastic surgery services had lower death rates, likely due to treating mostly minor burns or quickly transferring severe cases to specialized hospitals, reflected in their higher transfer rates compared to hospitals with plastic surgeons (29.2% vs. 6.01%).

The multivariable analysis revealed an important finding: after adjusting for TBSA and age, the association between facility type and mortality became stronger rather than weaker. Non‐BCU facilities showed even lower adjusted odds of death compared to BCUs (aOR 0.49 and 0.36 vs. crude OR 0.77 and 0.43). Notably, non‐BCU hospitals with plastic surgery treated the most severe burns by TBSA (mean 42.0% vs. 35.2% in BCUs) yet had lower mortality rates (7.78% vs. 9.87%). This paradox strongly suggests that BCUs receive patients with additional unmeasured risk factors beyond TBSA and age, such as inhalation injuries, comorbidities, or multi‐trauma. Furthermore, critically ill patients from non‐BCU facilities may be transferred to BCUs before death, creating referral bias. These findings support the interpretation that BCUs serve as the final destination for the most complex burn patients, whose higher mortality reflects case complexity rather than quality of care.

Another key finding concerns the stratification of mortality rates by burn severity. In line with previous literature, our results show that mortality rates increase sharply for burns ≥ 70% of TBSA, reaching 58.8% in facilities with plastic surgeons and 57.3% in BCUs. These findings are consistent with international data, where burns covering 80%–90% TBSA are associated with mortality rates ranging from 50% to 90%, depending on the availability of early excision and grafting, intensive care support, and infection control measures [1, 16].

The initial assessment of burn injuries based on the TBSA affected, guides initial resuscitation, patient admission, and the need for referral to a BCU. Several methods exist for estimating TBSA, the most well‐known being Wallace's “Rule of Nines,” however, it often overestimates burn severity in pediatric patients due to differences in anatomical proportionality between adults and children [17]. Alternatively, the Lund‐Browder system, routinely used in patients admitted to BCUs, accounts for more accurate age‐adjusted estimates [18], but its complexity can make it challenging to use in the field, especially due to unfamiliarity and limited training with burn assessment [19]. In view of this, the discrepancy between mortality rates observed in specialized and non‐specialized facilities for extensive burns raises concerns regarding potential inaccuracies in TBSA assessment in lower‐tier hospitals. As suggested by previous studies, prehospital providers frequently overestimate injury extent in both adult and children, leading to potential misclassification of burn severity and artificially lower mortality rates in secondary hospitals [20, 21].

### 4.1 Limitations

This study has some limitations that should be considered when interpreting the findings. One major issue is the reliability of the DATASUS database. Healthcare professionals in public hospitals within Brazil enter the data which may lead to inaccuracies and biases in routine practice [22]. Additionally, many procedures lack secondary diagnosis data, resulting in a high percentage of unspecified causes and decreased accuracy of the results. Importantly, the database does not include information on key prognostic factors such as inhalation injury, comorbidities (e.g., diabetes, cardiovascular disease), or validated burn severity scores (e.g., Revised Baux Score, Abbreviated Burn Severity Index). These unmeasured confounders likely contribute to the observed mortality differences between facility types and limit our ability to fully adjust for case complexity.

Another limitation is found in the way DATASUS organizes burn data. The database classifies burns by Total Body Surface Area (TBSA) but does not include information on burn depth. This means burns of different severities may be grouped together, affecting the accuracy of the analysis. As a result, treatment complexity may not be fully captured, leading to misclassification and oversimplification, especially when comparing hospitals with different levels of expertise. The inability to consider both TBSA and burn depth also weakens conclusions about patient outcomes. However, we used TBSA classification to simplify burn injury data and provide an overall picture of the quality of care.

The cross‐sectional design of this study prevents causal inference regarding the relationship between facility type and mortality. Referral bias is an important consideration, as critically ill patients may be transferred to BCUs before death, artificially inflating BCU mortality rates. Additionally, we could not capture patients who died during transfer or before reaching specialized care. The higher mortality at BCUs, even after multivariable adjustment, suggests residual confounding by unmeasured severity factors rather than differences in care quality.

We acknowledge that the “safe discharge” outcome aggregates various discharge categories (improvement, cure, discharge with follow‐up), which limits our ability to draw specific conclusions about the true effectiveness of care. Future studies with more granular outcome data would be valuable. Additionally, the definition of plastic surgery availability was based on CNES registry data, which may not fully capture the experience level or burn‐specific expertise of individual surgeons.

## 5 Conclusion

This study highlights the pivotal role of specialized burn care infrastructure and plastic surgery expertise in managing burn injuries in Brazil. Burn Care Units (BCUs) and facilities with plastic surgeons handled more severe cases, reflected in higher mortality rates, but demonstrated superior systemic outcomes, including reduced transfers and higher rates of safe discharge. A key finding was that non‐BCU hospitals with plastic surgery treated the most severe burns by TBSA (mean 42.0%) yet had lower mortality than BCUs (7.78% vs. 9.87%). Multivariable analysis confirmed that the higher mortality in BCUs persists after adjustment for TBSA and age, supporting the interpretation that BCUs receive patients with additional unmeasured risk factors (such as inhalation injury, comorbidities, or multi‐trauma) and serve as referral centers for the most complex cases. The presence of plastic surgeons was strongly associated with increased surgical intervention across all burn severities, underscoring their critical role in guiding evidence‐based care. In contrast, non‐specialized facilities relied disproportionately on conservative management, even for severe burns, likely reflecting resource limitations and gaps in specialized training, which may delay recovery or increase complication rates such as infection or scarring. These findings emphasize the need to expand access to surgical expertise and standardized protocols, particularly for severe burns, where early intervention improves survival.

Systemic challenges persist, including potential inaccuracies in burn severity assessment by non‐specialized providers, which may lead to misclassification and suboptimal triage. Strengthening training in TBSA evaluation, promoting multidisciplinary collaboration, and integrating structured referral pathways are essential to optimize burn care. By prioritizing surgical access, improving early intervention, and addressing disparities in expertise, Brazil can align its burn care system with global standards, reducing morbidity and mortality for all patients, regardless of injury severity.

## Author Contributions

**Paulo Henrique Moreira Melo:** conceptualization, data curation, formal analysis, methodology, writing – original draft, writing – review and editing. **João Oliveira Góes Neno:** data curation, writing – original draft, writing – review and editing. **Cynthia Florencio de Mesquita:** writing – review and editing. **Sarah Lopes Salomão:** writing – review and editing. **Lauren Kratky:** methodology, writing – original draft, writing – review and editing. **David P. Mooney:** conceptualization, supervision, writing – review and editing. **Cristina Pires Camargo:** conceptualization, supervision, writing – review and editing.

## Funding

The authors have nothing to report.

## Conflicts of Interest

The authors declare no conflicts of interest.

## Data Availability

The data that support the findings of this study are available in DATASUS at https://github.com/rfsaldanha/microdatasus. These data were derived from the following resources available in the public domain: ‐ microdatasus, https://github.com/rfsaldanha/microdatasus.
